# The immune-adjunctive potential of recombinant LAB vector expressing murine IFNλ3 (MuIFNλ3) against Type A Influenza Virus (IAV) infection

**DOI:** 10.1186/s13099-023-00578-5

**Published:** 2023-10-30

**Authors:** Sandeep Yadav, Aparna Varma, Aparna Odayil Muralidharan, Sucharita Bhowmick, Samiran Mondal, Amirul Islam Mallick

**Affiliations:** 1 Department of Biological Sciences, Indian Institute of Science Education and Research Kolkata, Mohanpur, Nadia, West Bengal 741246 India; 2 Department of Veterinary Pathology, West Bengal University of Animal and Fishery Sciences, Kolkata, West Bengal 700037 India

**Keywords:** Murine IFNλ3 (MuIFNλ3), *Lactococcus lactis* (*L. lactis*), Type A Influenza Virus (IAV), Immune protection

## Abstract

**Background:**

The conventional means of controlling the recurring pandemics of Type A Influenza Virus (IAV) infections remain challenging primarily because of its high mutability and increasing drug resistance. As an alternative to control IAV infections, the prophylactic use of cytokines to drive immune activation of multiple antiviral host factors has been progressively recognized. Among them, Type III Interferons (IFNs) exhibit a pivotal role in inducing potent antiviral host responses by upregulating the expression of several antiviral genes, including the Interferon-Stimulated Genes (ISGs) that specifically target the virus replication machinery. To harness the immuno-adjunctive potential, we examined whether pre-treatment of IFNλ3, a Type III IFN, can activate antiviral host responses against IAV infections.

**Methods:**

In the present study, we bioengineered a food-grade lactic acid-producing bacteria (LAB), *Lactococcus lactis* (*L. lactis*), to express and secrete functional murine IFNλ3 (MuIFNλ3) protein in the extracellular milieu. To test the immune-protective potential of MuIFNλ3 secreted by recombinant *L. lactis* (r*L. lactis),* we used murine B16F10 cells as an in vitro model while mice (BALB/c) were used for in vivo studies.

**Results:**

Our study demonstrated that priming with MuIFNλ3 secreted by r*L. lactis* could upregulate the expression of several antiviral genes, including Interferon Regulatory Factors (IRFs) and ISGs, without exacerbated pulmonary or intestinal inflammatory responses. Moreover, we also showed that pre-treatment of B16F10 cells with MuIFNλ3 can confer marked immune protection against mice-adapted influenza virus, A/PR/8/1934 (H1N1) infection.

**Conclusion:**

Since the primary target for IAV infections is the upper respiratory and gastrointestinal tract, immune activation without affecting the tissue homeostasis suggests the immune-adjunctive potential of IFNλ3 against IAV infections.

**Supplementary Information:**

The online version contains supplementary material available at 10.1186/s13099-023-00578-5.

## Background

Frequent outbreaks of Type A Influenza Virus (IAV) and their significant impact on human and animal health have warranted the strategic improvement of currently available control measures against IAV infections [1, 2]. Despite substantial advances in research towards vaccine-induced immune protection against IAV infections, most of these approaches fall short of efficacy in terms of enduring cross-protectivity or conferring long-term immune protection [3]. On the other hand, the growing resistance of IAVs to antiviral drugs such as oseltamivir, zanamivir, baloxavir, and peramivir has emerged as a major public health threat globally [4]. Moreover, these drugs are often associated with moderate to severe side effects, including nausea and bronchospasm, and their use is reportedly contraindicated for children and pregnant women [5–9]. In the search for effective measures to control IAV infections, recently exogenous administration of pro-inflammatory cytokines as an adjunct to vaccines or other therapeutic candidates has gained significant attention. Many cytokines, particularly interferons (IFNs), are known for their critical role in modulating innate and adaptive host responses against many viruses, including IAV infections [10–15]. The immune-modulatory role of IFNs was studied in depth against various subtypes of IAV infections, and the experimental use of Type I IFNs (IFNα) and Type II (IFNγ) were reported to be effective against influenza virus replication in multiple hosts [5–8, 12, 16]. Type I and Type II IFNs are mainly produced by the immune cells and can restrict virus replication by upregulating several Interferon-Stimulatory Genes (ISGs) [13–15]. However, their therapeutic usage often outweighs their clinical benefits because of hematological toxicity, exerting flu-like symptoms, elevated transaminases, fatigue, and nausea [9]. Specifically, the ability of Type I IFNs to trigger context-specific hyper-immune activation of host cells can lead to “interferonopathies,” which are detrimental to the host [17–20].

On the other hand, specific expression of its receptors, limited side effects, and early production have placed Type III IFNs as a superior and safer class of IFNs, particularly against respiratory virus infections [12, 13]. The key advantage of Type III IFNs, particularly IFNλ3, is the ability to configure its function in a highly regulated manner while exerting a pro-inflammatory effect on the mucosal surfaces. As a Type III IFN family member, IFNλ3 can also serve as the first line of defence, restricting virus spread at the epithelial barrier and providing antiviral host protection without exhibiting exaggerated pro-inflammatory responses. Moreover, IFNλ3 can utilize the distinct cellular mechanism by which it can skew the Th1/Th2 polarization to the Th1 phenotypes [12, 21, 22].

Considering these facts, the present study explored the potential of IFNλ3 as an adjunctive immunomodulatory agent against IAV infections. Given the sequence homology of human IFNλ3 and murine IFNλ3 (MuIFNλ3), including their respective receptors, for the present study, we used MuIFNλ3 and a mouse-adapted human IAV strain, A/PR/8/1934 (H1N1). Since most cytokines function in a local paracrine fashion, to minimize premature degradation, non-specific binding, and rapid clearance from the systemic reservoir, we employed bio-engineered Lactic acid-producing bacteria (LAB), *Lactococcus lactis* (*L. lactis*) to secrete recombinant MuIFNλ3 extracellularly directly at the host-mucosal interface [10, 23]. To engineer *L. lactis* cells as a preferred mode of mucosal delivery of MuIFNλ3, we utilized the Nisin Controlled Gene Expression (NICE) system, known for expressing the heterologous proteins in a tightly controlled manner [10, 23, 24]. Moreover, *L. lactis* cells (NZ9000) used in this study are classified as GRAS (Generally Recognized As Safe), hence considered safe for in vivo application. For assessing the functionality and antiviral potential of r*L. lactis* secreting MuIFNλ3, we specifically used murine B16F10 melanoma cells, which exhibit spindle-shaped and epithelial-like cell morphology. More importantly, B16F10 cells express receptors for various influenza virus subtypes and Type III IFNs (IFNλR1 and IL10Rβ) [25–27]. To see the effect of the exogenous application of MuIFNλ3, we pre-treated B16F10 cells with r*L. lactis* expressing MuIFNλ3 in a transwell plate system before infecting them with the A/PR/8/1934 (H1N1) virus. We found that MuIFNλ3 pre-treated cells can exhibit modest upregulation of several key cytokines and immunoregulatory gene expression, including Interleukin-6 (IL-6), MX Dynamin like GTPase-1 (MX1), Interferon Regulatory Factor-7 (IRF-7), and ISG-15. Further, we confirmed that MuIFNλ3 priming could also restrict virus replication and reduce the cytopathic effects of host cells infected with the A/PR/8/1934 (H1N1) virus.

Finally, to see the effect of mucosal delivery of live r*L. lactis* expressing MuIFNλ3 in the lung and intestinal tissue, we performed an in vivo mice study. For this, mice were mucosally (oro-nasally) administered with r*L. lactis* for three consecutive weeks, and the lung and intestinal tissue were collected for histopathological and transcriptional analysis of key immunoregulatory genes. Similar to in vitro observation, we recorded a selective upregulation of the MX-1, ISG-15, IRF-7, and IL-6 without exacerbated pulmonary inflammatory changes in the lung tissue. However, no such changes could be seen in the intestinal tissue.

Given that IRF family proteins are crucial in sensing viral RNAs, particularly in driving the expression of ISGs, our data suggest that the pre-treatment with MuIFNλ3 secreted by r*L. lactis* can enhance the promoter binding ability of IRFs to upregulate the expression of MX-1 and ISG-15 [12–15]. Together, we provide evidence of the immune-adjunctive potential of IFNλ3 without affecting tissue homeostasis by utilizing a live vector-based delivery platform as a possible biotherapeutic modality against IAV infections.

## Results

### Protein purification and functional characterization of recombinant MuIFNλ3 (rMuIFNλ3) protein

**Fig. 1:**
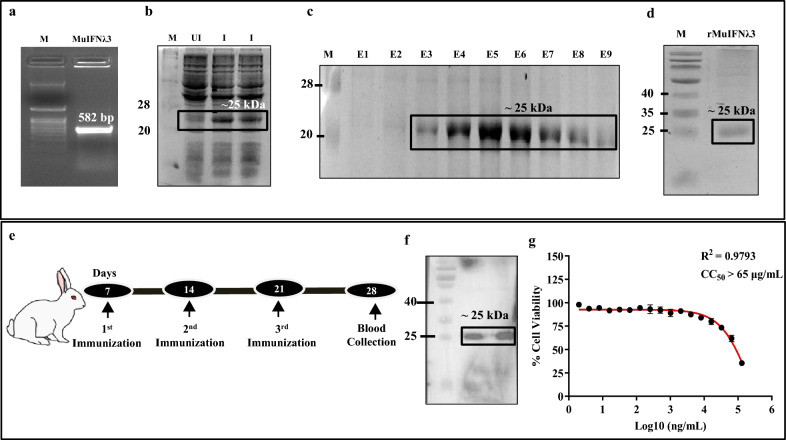
Cloning, expression, immunoreactivity, and cytotoxicity of rMuIFNλ3 protein expressed by *E. coli.* Colony PCR of the transformed *E. coli* BL21 (DE3) cells harboring pHis-MuIFNλ3 plasmid showing 582 bp product corresponding to the size of MuIFNλ3 gene (**a**). SDS PAGE analysis of IPTG-induced protein expression in the cell lysate of *E. coli* cells showing (closed box) the presence of rMuIFNλ3 protein with a molecular weight of ~ 25 kDa (**b**). Different elution fractions of Ni–NTA affinity column purified rMuIFNλ3 protein (E1 to E9, closed box) (**c**). Western blot analysis of purified His-tagged rMuIFNλ3 probed with mouse monoclonal anti-His antibody confirms the identity and size of the MuIFNλ3 protein (**d**). Schematic of the rabbit immunization schedule to raise hyperimmune sera against rMuIFNλ3 protein (**e**). Western blot analysis shows immunoreactivity of rMuIFNλ3 protein probed with rabbit hyperimmune sera. (**f**). Determination of CC_50_ of rMuIFNλ3 protein in murine B16F10 (65 μg/mL) (**g**). Each point represents the mean of three independent data sets ± standard deviation (SD) of the means

The colony PCR and gene sequencing confirmed the presence of target gene construct (rMuIFNλ3, 582 bp) in transformed *E. coli* BL21 (DE3) cells (Fig. 1a). The expression and purification of rMuIFNλ3 protein from recombinant *E. coli* cells were detected by SDS-PAGE analysis. The recombinant protein expressed by IPTG-induced *E. coli* cells was detected at ~ 25 kDa position, corresponding to the target protein size (Fig. 1b, c). Further, the protein identity and immunoreactivity were checked by immunoblot analysis using monoclonal anti-His antibody and rabbit polyclonal antisera raised against rMuIFNλ3 (Fig. 1d, f). The dose–response curve further suggests the safety profile of rMuIFNλ3 protein across two different murine cell types, B16F10 cells (CC_50_ value > 65 µg/mL; Fig. 1g) and J774A.1 cells (CC_50_ value > 100 µg/mL; Additional file 1: Fig. S1).

### Bioengineered r*L. lactis* showed stable expression of MuIFNλ3

**Fig. 2:**
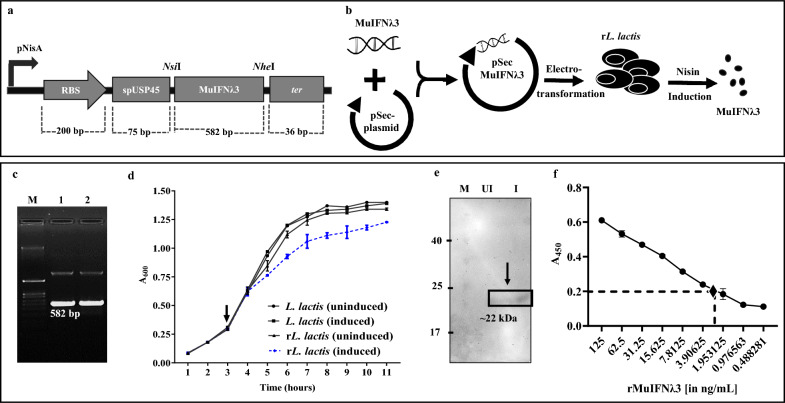
Construction of nisin-inducible MuIFNλ3 expression cassette and protein expression by r*L. lactis*. Recombinant plasmid expression cassette (pSec-MuIFNλ3) encoding signal peptide (sp) of lactococcal USP45 gene sequence (75 bp) followed by MuIFNλ3 gene (582 bp) flanked with *Nsi*I and *Nhe*I restriction sites (**a**). The graphical representation of the nisin-inducible expression and secretion of rMuIFNλ3 protein from r*L. lactis* cells (**b**). Agarose gel image showing specific amplification of rMuIFNλ3 gene with a size of 582 bp following colony PCR of electro-transformed *L. lactis* cells (NZ9000) (M: Marker; 1,2: r*L. lactis* colonies) (**c**). The comparative growth profile of WT *L. lactis*, nisin-induced or uninduced r*L. lactis* cells. Altered (reduced) growth profile of nisin-induced r*L. lactis* (dotted line) cells compared to uninduced r*L. lactis* or empty *L. lactis* (solid lines). “Arrow” indicates the time of nisin induction (**d**). Western blot analysis of TCA precipitated culture supernatant of nisin-induced r*L. lactis* cells probed with rabbit anti-rMuIFNλ3 antisera detect ~ 22 kDa protein corresponding to the size of MuIFNλ3 (**e**). Quantification of MuIFNλ3 protein secreted in the culture supernatants of r*L. lactis* by indirect ELISA suggests ~ 2.5 ng/mL of protein secreted by 1 × 10^9^ r*L. lactis* cells at 3 h post-induction. Dot “♦·” indicates the approximate quantification of protein secreted by induced r*L. lactis* (**f**)

Gene-specific PCR of the electro-transformed r*L. lactis* colonies and gene sequencing result of the expression cassette (pSec-SP_USP45_- MuIFNλ3) verified the presence of the MuIFNλ3 gene (582 bp) (Fig. 2c). The nisin inducibility (12 ng/mL) of the plasmid expressing MuIFNλ3 protein was affirmed by recording altered growth profiles of the r*L. lactis* cells compared to uninduced r*L.lactis* cells (OD_600_ difference of ~ 0.3–0.4) (Fig. 2d). However, no change was noticed in the case of induced or uninduced WT *L. lactis,* suggesting an increased metabolic burden in the recombinant bacterial cells.

Further, to validate whether the MuIFNλ3 protein is expressed by bioengineered r*L. lactis* secreted into the extracellular medium; the culture supernatant was TCA precipitated and subjected to Western blot analysis. Probing in the presence of rabbit polyclonal anti-mouse MuIFNλ3 antibody showed a specific band corresponding to the size of MuIFNλ3 protein (~ 22 kDa) (Fig. 2e). Compared to the protein standard, the amount of protein secreted by 1 × 10^9^ r*L. lactis* cells were estimated to be ∼ 2.25 ng after 4 h of induction with nisin (Fig. 2f, Additional file 1: Fig. S2a, b).

### Optimization of A/PR/8/1934 (H1N1) virus infection in murine B16F10 cells

**Fig. 3:**
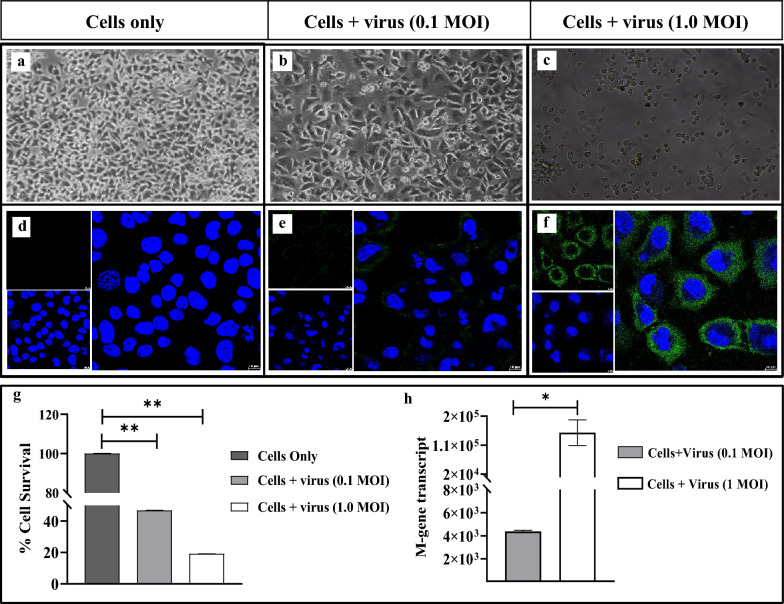
Optimization of A/PR/8/1934 (H1N1) virus infection in murine B16F10 cells. To optimize the viral infection in murine B16F10 melanoma cells, two MOIs (0.1 and 1.0) of the influenza A/PR/8/1934 (H1N1) virus were chosen. Virus infection was characterized by recording the visible CPEs characterized by rounding, swelling, and cellular detachment of virus-infected cells (**b**, **c**) compared to control cells (**a**). Virus-infected cells showing progressive accumulation of viral NP intracellularly (green fluorescence) (**e**, **f**, **d**). Images were captured in a Nikon TS100 inverted light microscope (Nikon, Tokyo, Japan) at ×20 magnification (**a–c**) and Leica SP8 confocal microscope using oil immersion ×63 objective; Scale Bar: 10 µm (**d–f**). Comparative analysis of percentage (%) of cell survival (**g**) and quantification of viral M-gene transcripts (**h**) in the cells infected with 0.1 and 1.0 MOI of the virus

To study the in vitro antiviral activity of MuIFNλ3, we chose murine B16F10 cells because of their ability to express IFNλ receptors and their permissivity to IAV infections due to their high lung tropism and replicative ability [25]. For optimal infection, viral infectivity of B16F10 cells was first checked by determining the 50% Tissue Culture Infectious Dose (TCID_50_/mL) of the virus stock and was found to be 5.7 × 10^3^/mL. Further, to establish the optimized infection in B16F10 cells, two different Multiplicity of Infection Doses (MOI) of the virus (0.1 and 1.0 MOI) were used. Based on the cell cytopathic effects (CPEs) scoring (rounding off cells and cellular detachment) and cell viability, we confirmed higher infectivity when 1.0 MOI of the virus was used (Fig. 3a–c, g). In terms of viral nucleoprotein (NP), both MOIs were found to elevate cytoplasmic accumulation of viral NPs (Fig. 3d–f). However, while checking for viral replication, infection with 1.0 MOI of the virus resulted in significantly higher M-gene transcripts compared to 0.1 MOI (Fig. 3h). Together, we confirmed the suitability of B16F10 cells for the IAV infection study.

### Pre-treatment with MuIFNλ3 upregulates the expression of immunoregulatory and antiviral genes in B16F10 cells

**Fig. 4:**
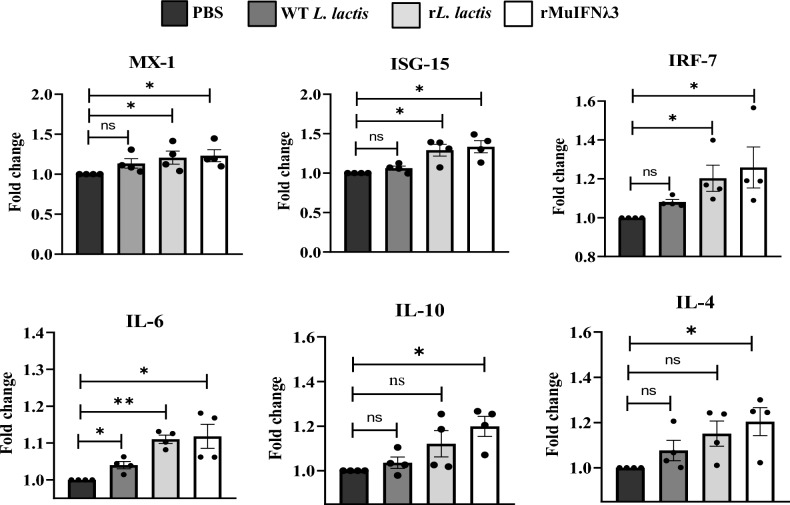
In vitro transcriptional profile of immune-regulatory and antiviral genes in response to MuIFNλ3 pre-treatment of B16F10 cells. Gene expression profiles of MX-1, ISG-15, IRF-7, IL-4, IL-6, and IL-10 genes were assessed in rMuIFNλ3 pre-treated murine B16F10 cells through semi-quantitative PCR. The total RNA was extracted from different treatment regimens: Cells only (no treatment, medium only), WT *L. lactis* (empty vector/NZ9000 cells), r*L. lactis* (nisin-induced), rMuIFNλ3 protein, and subjected to cDNA synthesis. The murine GAPDH gene served as an internal control. The data presented mean fold changes (compared to control cells) of four independent experiments performed under similar conditions. Asterisk (*) indicates the statistically significant difference, and “ns” indicates a non-significant difference

Pre-treatment of B16F10 cells with r*L. lactis* expressing MuIFNλ3 showed a significant increase in MX-1, IRF-7, ISG-15, IL-6, IL-4, and IL-10 gene expression; however, the fold changes were found to be within the range of 0.2 to 0.5 (compared to PBS and WT *L. lactis*). Here, in the case of rMuIFNλ3 and r*L. lactis* groups, marked upregulation of indicated MX-1, IRF-7, ISG-15, and IL-6 compared to the control groups (PBS and WT *L. lactis*) (Fig. 4). However, no visible changes were observed in the case of IL-4 among different experimental groups (Additional file 1: Fig. S3a, Table S1).

### MuIFNλ3 pre-treatment imparts in vitro protection against A/PR/8/1934 (H1N1) virus infection

#### Significant reduction of CPEs with higher cell survival

**Fig. 5:**
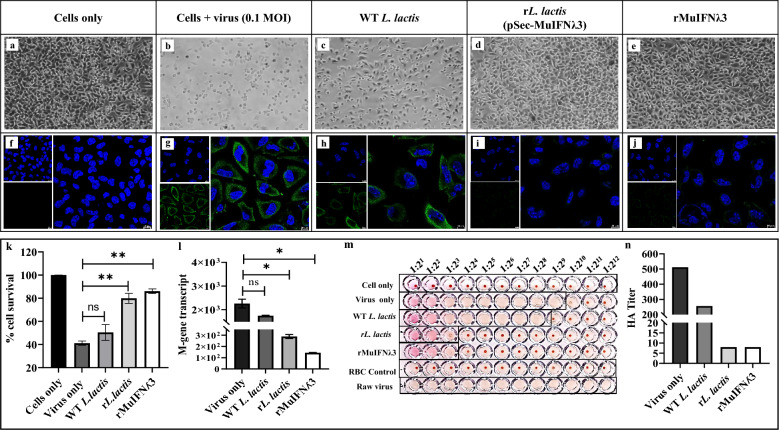
Effect of rMuIFNλ3 pre-treatment on B16F10 cells against A/PR/8/1934 (H1N1) infection. Representative images showing comparative CPEs exhibited by virus-infected B16F10 cells (1.0 MOI) pre-treated with different forms of MuIFNλ3. The images were captured in the Nikon TS100 inverted light microscope (Nikon) at ×20 magnification **(a–e)**. Indirect immunofluorescence assay (IFA) showing a significant reduction of viral NP (in green) in the cells pre-treated with MuIFNλ3 secreted by r*L. lactis* or expressed by *E. coli* (**i**, **j**) compared to untreated/WT *L. lactis* treated infected cells (**g**, **h**). For nuclear staining 4′,6-diamidino-2-phenylindole-dihydrochloride (DAPI) (in blue) was used. All images were visualized in the Leica SP8 confocal microscope using oil immersion ×63 objective (NA 1.4), DAPI, and FITC filter. Blue fluorescence corresponds to DAPI staining of the nucleus, and green fluorescence corresponds to the H1N1 viral NP protein. Scale Bar: 50 µm (**f**, **j**). Percentage of cell survival by standard MTT assay showing significantly higher cell survival when cells were pre-treated with different forms of MuIFNλ3 (r*L. lactis* or rMuIFNλ3) against H1N1 virus infection. Each bar represents the mean of percent cell survival ± SE of three independent experiments performed in triplicates. Asterisks (**) in (*p* < *0.01*) indicate statistically significant differences (control vs. treatment) (**k**). Absolute quantification of viral M-gene transcripts by RT-qPCR showing substantial reduction in M-gene copies in infected cells pre-treated with MuIFNλ3 (r*L. lactis* and rMuIFNλ3) compared to the control groups (Virus only and WT *L. lactis*). Each bar represents the mean of the M-gene transcript number ± SE of two independent experiments performed in duplicates. Asterisks (*) indicate statistically significant differences *(p* ≤ *0.05)* with respect to the control (**l**). Viral haemagglutination (HA) assay to detect the virus titer in the culture supernatant of infected B16F10 indicates a significant reduction in HA titer in MuIFNλ3 pre-treated cells compared to controls. Two-fold serial dilutions of each sample were incubated with 0.5% chicken RBC (cRBC). The reciprocal of the highest dilution of the sample that causes complete hemagglutination of cRBC was considered HA titer (**m**). Each bar represents the HA titer of different treatment setups and is compared to the control, showing a significant reduction in HA titer in the case of MuIFNλ3 treated cells (**n**)

To assess the protective efficacy of MuIFNλ3, B16F10 cells were infected with 1.0 MOI of the virus. After 24 h post-infection, we observed a significant reduction of CPEs in terms of cell swelling, rounding, and detachment in cells pre-treated with different forms of MuIFNλ3 (Fig. 5a–e) compared to control cells (virus-only group and WT *L. lactis*). Further, we performed MTT assay to quantify the percentage of cell survival. It was found to be significantly higher in the case of MuIFNλ3 pre-treatment, suggesting an effective inhibition in virus replication (Fig. 5k).

#### Decreased intracellular accumulation of viral NPs and viral M-gene transcripts

A marked reduction in fluorescence signal in the infected cells (pre-treated with MuIFNλ3) compared to untreated virus-infected cells indicates a significant decrease in cellular accumulation of viral NP (Fig. 5f–j).

To see the effect of MuIFNλ3 on viral replication, viral M-gene transcripts were quantified by RT-qPCR. Compared to the controls (virus-only group and WT *L. lactis*), a significantly low copy number of viral transcripts suggests blocking viral replication in the rMuIFNλ3 or r*L. lactis* pre-treated groups (Fig. 5l).

#### Reduction in viral HA titer

To assess the residual viral load, the culture supernatant of B16F10 cells was titrated for viral HA antigen. The data indicate a significant reduction in HA titer in the case of both rMuIFNλ3 and r*L. lactis*-treated cells in comparison to the control groups (untreated infected cells or treated with WT *L. lactis*) (Fig. 5m, n).

### Fecal retrieval of r*L. lactis* cells harboring intact plasmid

The fecal contents from each of the mice from the respective groups were plated in GM17 plates supplemented with 0.5% glucose and with or without 20 μg/mL chloramphenicol. After incubation at 30 °C overnight, in the case of the r*L. lactis*-treated groups, many colonies appeared, and no colonies were observed in the plates supplemented with 20 μg/mL chloramphenicol for the WT *L. lactis* group (Additional file 1: Fig. S4a–c). Colony PCR using randomly picked colonies from these plates using gene-specific primers revealed the presence of intact plasmids with the MuIFNλ3 gene constructs, confirming the intragastric stability of *L. lactis* and its capacity to keep plasmid intact (Additional file 1: Fig. S4d).

### Transcriptional analysis of immunoregulatory and antiviral genes in lung and intestinal tissue of experimental mice

**Fig. 6:**
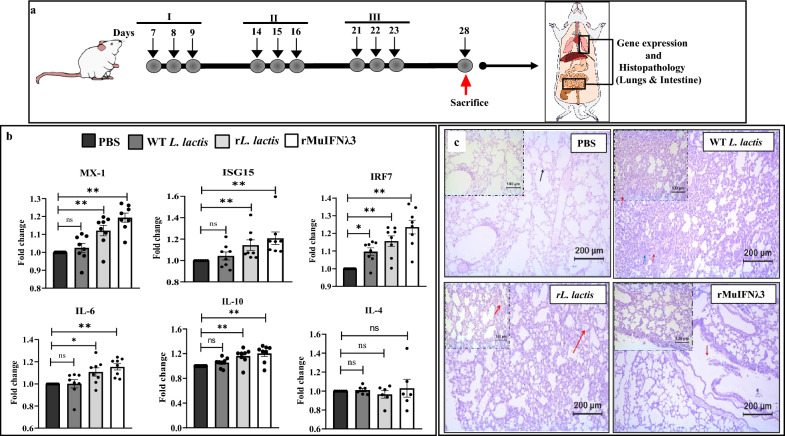
In vivo mice study for oro-nasal administration of rMuIFNλ3. Schematic of in vivo mice study for oro-nasal administration of rMuIFNλ3 in different forms (**a**). Gene expression profile in lung tissue of mice showing differential expression of MX-1 (upregulated), IRF-7 (upregulated), ISG-15 (upregulated), and IL-6 (upregulated). Bar indicates the fold changes of genes with respect to control groups of mice (received PBS only). Asterisk (*) indicates the statistically significant difference, and “ns” indicates a non-significant difference (**b**). Representative images of Hematoxylin and Eosin (H & E) stained lung tissue sections from experimental mice. The images of the lung tissues of the WT *L. lactis* group specify the alveolar and bronchiolar epithelial hyperplasia (blue and red arrow). Thickening of the alveolar septa and the accumulation of the oedematic fluid in the alveolar space with mononuclear cell infiltration seen as pink-colored space with cells (red arrow) was observed in the case of *rL. lactis*. The tissues from the rMuIFNλ3 group had necrotic and degenerative changes (red arrow) in the terminal bronchial. All stained tissue sections were digitally scanned under a ×20 objective lens (Leica DM 2000 LAe). Scale bars: 100 μm (**c**)

The expression profile of various immunoregulatory and antiviral genes was studied in the lung and intestine tissues collected from each treatment group. In the case of lung tissue, data suggest a marked increase in the expression of MX-1, ISG-15, IRF-7, IL-6, and IL-10 in the mice that received different forms of MuIFNλ3 protein compared to the control groups (Fig. 6b, Additional file 1: Fig. S3b, Table S1). However, we did not observe such increased expression of genes in intestine tissue among various groups (Additional file 1: Fig. S5b, Table S1).

### Histopathological analysis of lung and intestinal tissue

#### Lungs

The lung tissue sections from mice of different experimental groups show mononuclear cell infiltration with visible thickening of alveolar septa and accumulation of some oedematic fluid following oro-nasal administration of live r*L. lactis* secreting MuIFNλ3 (Fig. 6c). The migration of monocytes to the lungs suggests a temporal role of IFNλ3 in recruiting monocytes to lung epithelial cells, the critical site for viral clearance. Although mice receiving WT *L. lactis* showed bronchiolization and signs of hyperplasia in bronchiolar and alveolar epithelial cells, no significant mononuclear cell infiltration was found. In contrast, mice that received an injectable form of IFA conjugated rMuIFNλ3 protein showed a transition of alveolar cells from squamous to cuboidal type with some necrotic changes in the terminal bronchi. The apical portion of the epithelia showed blebbing out to the lumen, along with mild fibrotic changes noted around the bronchiole. As expected, untreated mice exhibited visible airspace, with the alveoli surrounded by squamous epithelial cells and no changes in the vascular endothelial lining. There was no vasocongestion, and the terminal bronchiole appeared to be lined with simple cuboidal epithelial cells.

#### Small intestine

Comparative analysis of histopathological changes observed in the intentional section of the different experimental groups of mice indicate no noticeable change except for the mice that received systemic (s/c) administration of rMuIFNλ3 protein (Additional file 1: Fig. S5a). Mice injected with IFA-emulsified MuIFNλ3 exhibited multi-focal mononuclear infiltration in the submucosal layer and disruption in the epithelial layer of the villi and the outer muscular layers. The critical observation pointed out minor congestion of the blood vessels with low-grade hyperplasia of the outer longitudinal and inner circular muscle layer in r*L. lactis* administered mice. Moreover, circular folds of villi lining the columnar epithelium in the mucosal layer suggest normal tissue architecture. In the WT *L. lactis* group*,* minor mononuclear cell infiltration with mild congestion of blood vessels in the sub-mucosal layer and slight degenerative changes in the columnar epithelium were observed.

## Discussion

The complex network of cytokines and other immunoregulatory factors that trigger innate or specific immune responses against viral infections are primarily mediated by the recruitment and activation of effector immune cells [28, 29]. However, with the intrinsic ability of viruses to interfere with cytokine-induced signaling pathways, most of them can alter downstream effector functions, facilitating their self-survival and replication [30, 31]. Alternatively, dysregulated pro-inflammatory responses due to some viral infections, including IAV, can compromise host defense by resulting in moderate to severe tissue damage, commonly known as cytokine storm [22, 32]. However, the key mediators for such heightened immune responses are also part of a well-conserved innate immune response necessary for the efficient clearance of viruses. Gaining a deeper understanding of the pleiotropic role of cytokines can help distinguish cytokine-driven physiological consequences from their pathophysiological outcomes. This information can lead to the refined and safe use of cytokine as a biotherapeutic candidate against several viral infections.

To this end, the targeted use of immunoregulatory proteins has attracted significant attention to counter virus-induced cytokine storms. One of the most rational and coherent approaches to mitigate this is the tailored application of cytokine targeting specific infection processes, such as virus replication machinery. Clinical and experimental studies have perceived the successful use of pro-inflammatory cytokines primarily as an immune adjuvant against many viruses, including IAV infections [12, 22, 27, 33–36]. However, in addition to the risk of inducing an exaggerated immune response, pro-inflammatory cytokines also suffer from their poor pharmaco-kinetics, short half-life, low pharmacological doses, and the risk of systemic toxicity. This entails careful selection of the cytokine class, dosage, and the appropriate mode of delivery [37, 38]. Our previous study reported that pre-treatment of chicken interleukin-17A (ChIL-17A) secreted by a modified LAB vector could induce moderate immune protection against IAV infections in chicken cells [10]. However, as a potent pro-inflammatory cytokine, IL-17A often promotes aggravation of symptoms, acute lung injury, and tissue damage at the epithelial barriers during infections [39–41].

Advances in understanding the mechanism of the pro-inflammatory function of the IFN system suggest that IFNs possess unique immunoregulatory activities that significantly mediate antiviral host defense against IAV infections [42–45]. Among the different classes of IFNs, Type II IFNs are elevated considerably during influenza infection; hence, their use as cytokine-based therapeutic interventions is uncertain [46, 47]. As an emerging alternative, several studies in the recent past endorsed the applicability of a different IFN class, such as Type III, in modulating host responses to respiratory virus infection [25]. Published reports further indicate that among the members of the Type III IFN family, IFNλ3, in comparison to its other counterparts (IFNλ1 and IFNλ2) and other families of interferons (IFNα, IFNβ, IFNγ, etc.), can impart potent antiviral activities without intense inflammatory effects [21, 22, 48]*.* Considering the selective receptor expression on non-immune cells, we hypothesized that oro-nasal application of IFNλ3 may collectively induce a controlled immune response after binding to its specific receptors on mucosal epithelial cells and show an elevated antiviral state in the host [49].

To this end, we aimed to study the effect of the exogenous application of rMuIFNλ3 first in an in vitro setup and then in in vivo mice models. For this, we bioengineered *L. lactis* cells to express and secrete functionally bioactive MuIFNλ3 and showed the extended stability of r*L. lactis* cells harbouring MuIFNλ3 during gut transit (Additional file 1: Fig. S4a–d). Moreover, using the NICE system, we could show stable expression of MuIFNλ3 in the culture supernatant. The NICE system is efficient for protein secretion as it expresses the signal peptide (SP) of the lactococcal-secreted protein USP45 (spUSP45). We presented efficient protein secretion utilizing the spUSP45, as evident from the successful detection of MuIFNλ3 protein in the culture supernatant [50].

To evaluate the immunological correlates of MuIFNλ3 pre-treatment, we used murine B16F10 melanoma cells known for constitutive expression of IFNλR and highly permissive to IAVs. Using these cells, we demonstrated that pre-treatment with IFNλ3 expressed by r*L. lactis* could elicit elevated expression of MX-1, ISG-15, IRF-7, and IL-6 genes and impart significant immune protection against IAV infections. Similar to the in vitro study, we also observed marked expression of these genes in the lung tissue of mice that received oro-nasal administration of r*L. lactis* secreting MuIFNλ3 (Fig. 6b).

The binding of IFNλ3 leads to the dimerization of the receptors (IL-28AR and IL-10R2), and the subsequent JAK transphosphorylation leads to nuclear translocation of the ISGF3 complex. The elevated expression of MX-1, ISG-15, IRF-7, and IL-6, following IFNλ3 priming, suggests effective activation of the JAK-STAT pathway to trigger a downstream cascade effect by releasing ISGs and thus inhibition of virus replication [51, 52]. Since IRFs play a significant role in cytosolic nucleic acid sensing and the endosomal Toll-like receptor (TLR) signaling, it would be interesting to see how IFNλ3 pre-treatment modulates innate sensing and signaling of IAV infections by engaging these IRFs [53].

Intriguingly, we noted some upregulation of IL-10 gene expression in vitro and in vivo experiments. Although structural similarities exist between Type III IFNs and IL-10 family cytokines and their respective receptors, whether the observed increase in IL-10 is due to the consequence of the shared IL10Rβ chain on interactions between IFNλ and IL-10 receptors is unclear [54]. Because both use shared receptor (IL10R2), IL-10 expression may also contribute to negative feedback control of IFNλ3 effector function [55].

While not tested, increased IL-6 gene expression suggests T-cell response regulation, macrophage migration, and phagocytic activations, ensuring effective antiviral host responses [11]. Moreover, given that Th1/Th2 to Th1 polarization is an intrinsic property of IFNλ3 signaling cascade, a moderate increase in IL-4 expression observed in this study could presumably be due to increased expression of IL-6 as an indirect effect of IFNλ3 signaling cascade [56, 57].

Interestingly, similar to the in vitro study, we observed marked upregulation of MX-1 genes in the lungs of mice that received oro-nasal administration of r*L. lactis*. As an evolutionarily conserved dynamin-like large GTPases, Mx-1 protein, through its GTPase activity, drives antiviral activities by suppressing the polymerase activity of viral ribonucleoproteins (vRNPs) [58]. Emerging evidence suggests that the expression of the MX-1 gene is directly regulated by Type I and III IFNs [58–61]. However, most of the commonly used laboratory mouse strains, including C57BL/6 or BALB/c mice, have lost the expression of functional Mx-1 protein due to the absence of 424 nucleotides from the coding region of the MX-1 mRNA [14, 15]. Therefore, elevated expression of MX-1 cannot be linked to the observed protection; hence, future studies are required to use an appropriate model that expresses functional Mx-1 protein.

Nevertheless, when checked for protection against H1N1 infection in vitro, we observed a significant reduction in CPEs with a marked reduction in intracellular accumulation of viral NPs in the cells pre-treated with MuIFNλ3. This observation was further verified by the low copy number of viral M-gene transcript in the treated cells. Finally, to see the effect of oro-nasal administration rLAB vector expressing MuIFNλ3 on lung homeostasis, except for profound infiltration of mononuclear cells, no noticeable change could be observed in the mice that received mucosal administration of the r*L. lactis* cells. However, when checked for the systemic administration group, some necrotic and degenerative changes could also be seen in addition to mononuclear infiltration. Further challenge experiments are required to determine whether the observed inflammatory response in the lungs can facilitate viral clearance without excessive inflammation and tissue damage (Fig. 6c). On the other hand, mice that received NZ9000 cells (WT *L. lactis*) show mild hyperplasia in the alveolar and bronchiolar epithelial cells. Together, histopathological analysis of lung tissue shows direct evidence of superior therapeutic outcomes when MuIFNλ3 is delivered via live r*L*. *lactis* cells. Except for IFA- rMuIFNλ3 injected mice, no visible differences could be observed in the intestinal tissue, possibly due to limited tissue availability of MuIFNλ3 secreted by r*L. lactis* (Additional file 1: Fig. S5a).

Based on these results, our study illustrates how exogenous application of IFNλ3 using a live vector-based delivery platform can modulate the host immune responses and augment the antiviral state in the host. Therefore, we propose that Type III IFNs, IFNλ3 in particular, have excellent bio-therapeutic potential with broad spectrum activity against emerging respiratory viruses.

## Conclusion

The conventional vaccines or antiviral approaches against currently circulating IAVs are highly subtype-specific and have multiple downsides. As an alternative means to control IAV infections, we demonstrated the immune-adjunctive potential of IFNλ3 against IAV infections using a live vector-based delivery platform. We discussed the potential of the current strategy in augmenting the host antiviral state, implying the benefits of the mucosal administration of IFNλ3 as an alternative to conventional approaches.

## Methods

### Bacterial strains, plasmids, and growth conditions

The bacterial strains and plasmids used in the present study are listed in Table 1. *E. coli* BL21 (DE3) cells were cultured in the Luria Bertani (LB) Media (Himedia, India) under continuous shaking at 37 °C. The *L. lactis* (NZ9000) cells were cultured in M17 media (Himedia, India) supplemented with 0.5% (w/v) glucose (Merck, Germany) at 30 °C under static conditions.

**Table 1:** List of the bacterial strains, plasmids, and viruses used in this study

Bacterial strains, plasmids and viruses	Details	Purpose	Source
pHis-TEV	Amp^r^, 6X-Histidine tag (N-terminal), T7 promoter, TEV-tag and cleavage site	Expression vector	BioBharati LifeSciences, India
pHis-MuIFNλ3	Amp^r^, 6X-Histidine tag (N-terminal), TEV-tag and cleavage site, T7 promoter in plasmid backbone. Harbours the MuIFNλ3 gene sequence	Expression of recombinant Murine-IFNλ3 protein	This work
pSec-MuIFNλ3	Cm^r^, spUSP45, pNisA promoter, trpA terminator in plasmid backbone, harbouring the MuIFNλ3 gene sequence	Expression of recombinant Murine-IFNλ3 in *L. lactis*	This work
*E. coli* BL21 (DE3)	F– ompT Ion hsdSB (rB–, mB–) gal dcm (DE3)	Expression host for recombinant proteins	BioBharati LifeSciences
*L. lactis* (NZ9000)	MG1363 (nisRK genes into chromosome), Wild type plasmid free	Wild type bacteria (control)	Dr. Luis G Bermúdez Humar´an, French National Institute for Agricultural Research, Paris, France
r*L. lactis* (rNZ9000)	MG1363 (nisRK genes into chromosome), harbouring the pSec-MuIFNλ3 plasmid	Expression host for recombinant proteins in the secretory form	This work
Influenza A Virus (H1N1)	A/PR/8/1934(H1N1) (VR-95™)	To infect murine B16F10 cells (IBSC approval no. IISERK/IBSC/2019/005)	ATCC, USA

### Virus and cell culture

The murine melanoma cells (B16F10 cells) were purchased from NCCS (Pune, India) and murine macrophage cells (J774A.1) were procured from ATCC (USA). Both cells were maintained in complete Dulbecco’s Modified Eagle’s medium (DMEM) (Invitrogen, USA), supplemented with 10% fetal bovine serum (FBS) (Gibco, USA), 100 U/mL Penicillin, and 100 μg/mL Streptomycin (P/S) (HiMedia) under 5% CO_2_ at 37 °C.

The Influenza Type A/PR/8/1934 (H1N1) virus was purchased from ATCC (ATCC ® VR95 ™) and propagated in embryonated chicken eggs as per the published method with some modifications in our lab [10]. Briefly, 10^–2^ dilution of the virus stock was propagated in embryonated 10–11 days-old chicken eggs for 48 h at 37 °C under 60% humidity. The allantoic fluid was subjected to viral titer determination by haemagglutination assay and TCID_50_ /mL value estimation as per the published methods [10, 62, 63].

### Cloning, expression, and purification of rMuIFNλ3 in *E. coli*

The pCMV3 untagged vector containing the full-length cDNA clone of MuIFNλ3 (IL28B) was procured from Sino Biological Inc., Japan (Catalog No: MG51306-UT). The target gene sequence (582 bp) was PCR amplified from the vector and cloned into pHis-TEV expression vector (BioBharti, India), having an N-terminal 6X-Histidine tag in-frame, and subsequently transformed into chemically competent *E. coli* BL21 (DE3) cells (BioBharati, India). The expression of the recombinant MuIFNλ3 protein was optimized by induction with 1.0 mM IPTG (Isopropyl ß-d-1-thiogalactopyranoside) (Sigma Aldrich, USA) when OD_600_ of seed culture reached ~ 0.4–0.5. Induced *E. coli* cells were further incubated for 6–7 h under continuous shaking at 37 °C. The cells were then collected after centrifugation at 5000×*g* at 4 °C, and the cell pellet was resuspended in 10 mL cell lysis buffer (6 M Guanidine hydrochloride, 50 mM NaH_2_PO_4,_ and 300 mM NaCl; pH ~ 8.0). The resuspended pellet was sonicated with 5 s pulses at 35% amplitude and an intermediate stop of 10 s for 10–15 min (Sonics and Materials Inc., USA). The supernatant of the lysate was collected by centrifugation at 15,000×*g* for 10 min at 4 °C and the supernatant was subjected to Ni- NTA column chromatography to purify the His-tagged recombinant protein as per manufacturer’s instruction (Qiagen, USA). The eluted protein fraction was dialyzed, and the protein concentration was measured by the bicinchoninic acid (BCA) method using a commercial kit (Thermo Fisher Scientific, USA). The size and the purity of the protein were further confirmed by SDS-PAGE as well as by Western blot analysis using mouse monoclonal anti-His antibody (Thermo Fisher Scientific, USA). For Western blot analysis, the protein was transferred to PVDF (Polyvinylidene fluoride) membrane and blocked overnight with 3% BSA and probed with mouse monoclonal anti-His antibody (1:5000) for 1 h at room temperature (RT). The membrane was washed thrice with TBS (50 mM Tris–Cl, 150 mM NaCl, pH 7.5) and twice with TBS-T (TBS with 0.1% Tween-20) solution for 5 min each and then incubated with horseradish peroxidase (HRP)-conjugated goat anti-mouse IgG (H & L) secondary antibody (1:5000) (Thermo Fisher Scientific, USA) for 1 h at RT. Finally, 3,3′-Diaminobenzidine (DAB) solution (Sigma Aldrich, USA) was used as a substrate to develop the blot.

### Assessing the immunoreactivity of rMuIFNλ3 protein

To assess the immunogenicity of rMuIFNλ3, New Zealand White rabbits were used to raise polyclonal antibodies as per the standard protocol described elsewhere [10]. Briefly, 100 μg of rMuIFNλ3 protein emulsified in Complete Freund’s Adjuvant (CFA) (Sigma Aldrich, USA) was administered on day 7 (Fig. 1e), followed by secondary immunization on days 14 and 21 with 50 μg of rMuIFNλ3 protein emulsified in Incomplete Freund’s Adjuvant (IFA) (Sigma Aldrich, USA). Seven days after the last immunization, blood was collected from the marginal ear veins, serum was separated, and immunoreactivity against rMuIFNλ3 protein was checked by Western blot analysis.

### Assessing the in vitro cell cytotoxicity of purified rMuIFNλ3 protein

To determine the cytotoxicity of the purified rMuIFNλ3 protein, we performed the standard MTT [3-(4, 5-dimethylthiazol-2-yl)-2, 5-diphenyl tetrasodium bromide] assay in murine B16F10 cells and J774A.1 cells as described previously with minor modifications [64]. In brief, cells were grown in DMEM media containing 10% FBS and 1% P/S and seeded at a density of 1 × 10^4^ cells in a 96-well tissue culture plate (Thermo Fischer Scientific, USA) and allowed to grow up to more than 80% confluency. Further, the cells were treated with rMuIFNλ3 protein starting with a concentration of 130 µg/mL and incubated for 24 h at 37 °C under 5% CO_2_. Following incubation, cells were then washed with 1 X PBS, and 100 µL MTT solution (0.1 mg/mL in DMEM) was added to each well and incubated at 37 °C under 5% CO_2_ for 4 h. The formazan crystals (MTT metabolic product) thus formed were dissolved in 100 µL Dimethyl sulfoxide (DMSO), and the absorbance was (A595 nm) in Epoch 2 spectrophotometer (Biotek). Finally, the 50% cytotoxic concentration (CC_50_) was determined from the dose–response curve (percentage cell survival vs. protein concentration).

### Bioengineering *L. lactis* cells expressing rMuIFNλ3

The MuIFNλ3 gene sequence was PCR-amplified from the pCMV3 vector and cloned into a nisin-inducible pSec plasmid backbone in-frame with an N-terminal signal peptide sequence (spUSP45). The schematic of the cloning strategy and protein expression is provided in Fig. 2a, b. The modified pSec plasmid encoding the MuIFNλ3 gene sequence was then transformed into a food-grade LAB vector of *L. lactis* sub sp. *cremoris* (strain NZ9000) by electroporation. The recombinant *L. lactis* (r*L. lactis*) colonies that appeared on M17 agar plates supplemented with 0.5% (w/v) glucose and 20 µg/mL chloramphenicol were screened by colony PCR using a gene-specific primer set, followed by gene sequencing.

### Detection of MuIFNλ3 protein in the culture supernatant of r*L. lactis*

The selected clones were tested for nisin inducibility using varied concentrations of nisin (1–15 ng/mL), induction time, and the effect on bacterial growth [10, 23]. Using optimized nisin concentration (12 ng/mL), the culture supernatant was harvested at 3 h post-induction by centrifuging the cells at 5000×*g* for 10 min. Ice-chilled Trichloroacetic acid (TCA) (5% w/v) (Merck) was added to the supernatant and incubated overnight at 4 °C. The precipitated protein pellet was then washed with chilled acetone (80% v/v), air-dried, and resuspended in IEF buffer [8 M Urea and 0.4 (w/v) dithiothreitol (DTT)] and subjected to Western blot analysis using polyclonal rabbit anti-MuIFNλ3 antibody (1:500 dilution) as primary antibody and HRP-conjugated goat anti-rabbit IgG (H & L) as the secondary antibody (1:2500 dilution) (Thermo Fisher Scientific, USA).

### Quantification of MuIFNλ3 secreted by r*L. lactis* culture

Indirect ELISA was performed to quantify the MuIFNλ3 protein secreted by the nisin-induced r*L. lactis* cells as described previously [10, 23]. Briefly, the TCA precipitated protein pellet from 100 mL culture supernatant of both nisin-induced and uninduced r*L. lactis* cells were reconstituted in PBS buffer and coated onto a 96-well ELISA plate (Nunc, USA) using carbonate-bicarbonate buffer (pH ~ 9.6) overnight at 4 °C. The plate was then washed thoroughly with PBS-T (0.05% Tween-20 in PBS) and blocked with 5% BSA at 37 °C for 1 h. Next, probing was done by adding 100 μL of rabbit polyclonal anti-MuIFNλ3 antibody (1:500 dilution) to each well and incubating for 2 h at RT, followed by washing and incubating with HRP-conjugated goat anti-rabbit IgG (H & L) secondary antibody (1:2500 dilution) (BioBharati, India) for 1 h at RT. Finally, the plate was washed thrice with PBS-T, followed by the addition of 200 µL of 3,3′,5,5′-tetramethylbenzidine (TMB) substrate to each well. Finally, the reaction was stopped by the addition of 50 µL stop solution [1 M sulphuric acid (Merck)] in each well. The absorbance was measured at 450 nm in Epoch 2 microplate reader (BioTek). A varied concentration of purified rMuIFNλ3 protein (1 to 1000 ng) expressed by *E. coli* was used as a standard for protein quantification [10, 23].

### Assessing the immunoregulatory effect of MuIFNλ3 pre-treatment of B16F10 cells

**Fig. 7:**
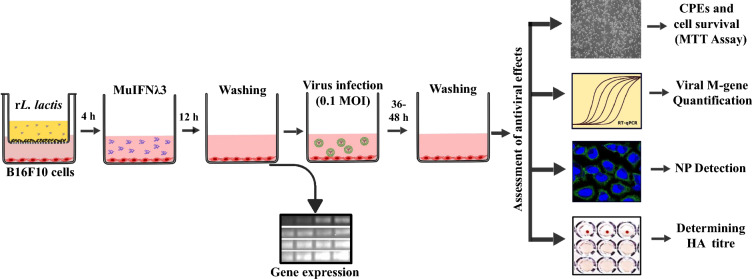
Schematic of in vitro experimental setup using a transwell system. Representative assay setup in transwell system showing murine B16F10 cells grown in bottom wells and nisin-induced r*L. lactis* cells were added in the upper inserts. After 4 h, cells were washed and incubated further for 12 h followed by infection with the virus (1.0 MOI). Residual infectivity of the virus was determined by cell survivability assay/CPE observation, M-gene quantification, presence of viral NP, and HA assay. In addition, before virus infection, the expression of various antiviral genes and cytokines was checked

To study the functionality and immunoregulatory effect of MuIFNλ3, murine B16F10 cells were treated with different forms of MuIFNλ3 protein. For *E. coli* purified rMuIFNλ3, the purified protein was directly added to the cells (1000 ng/mL) while treating the cells with MuIFNλ3 secreted by r*L. lactis*, we used a transwell plate module (Merck) according to the previously published method [10] (Fig. 7). Briefly**,** B16F10 cells (5 × 10^4^ /well) were seeded in a 24-well plate with 1 mL of complete DMEM and incubated for 24 h at 37 °C under 5% CO_2_. The r*L. lactis* cells were grown until OD_600_ reached ~ 0.3 to 0.5, followed by centrifugation at 2400×*g* for 5 min. The cell pellet was then resuspended with fresh growth media containing nisin (12 ng/mL) and added to the upper chamber of the transwell setup. Following incubation for 4 h at 37 °C, the upper chamber was removed, and cells in the bottom compartment were kept for 12 h at 37 °C under 5% CO_2_ in complete DMEM. The untreated cells [treated with 1X PBS or wild-type (WT) *L. lactis*] cells served as controls. Next, the cells were washed, and total RNA was extracted from cells using the TRIzol method according to the manufacturer’s instructions (Invitrogen, USA), and cDNA was synthesized using the Superscript MuLV cDNA Synthesis Kit (BioBharati, India) with oligo-dT primers, following the manufacturer’s protocol. The transcriptional analysis of murine MX-1, ISG-15, IRF-7, IL-6, IL-4, and IL-10 was performed by semi-quantitative PCR analysis, using MuGAPDH as an endogenous control. The details of the specific primers used for target gene amplification are provided in Table 2.

**Table 2:** List of the primers used for cloning and gene expression in this study

Target gene	Purpose	Primer sequence	Amplicon size (bp)	Annealing temperature (°C)	Source
MuIFNλ3	Cloning into pHis-TEV Plasmid	F-5′ATAGGATCCATGCTCCTCCTGCTG 3′ R-5′ATAAAGCTTTTATCAGACACACTGGTCTC 3′	582	60	This study
MuIFNλ3	Cloning into pSec Plasmid	F-5′ ACTATGCATCAGAGATGCTCCTCCTGCTGTT 3′ R-5′GATGCTAGCTTATCAGACACACTGGTCTCC 3′	582	59	This study
MuGAPDH	Semi-quantitative PCR	F-5′CGTGCCGCTGGAGAAACC 3′ R-5′TGGAAGAGTGGGAGTTGCTGTTG 3′	150	60	This study
MuMX-1	Semi-quantitative PCR	F-5′GAAGGCAAGGTCTTGGATG 3′ R-5′GCTGACCTCTGCACTTGACT 3′	82	60	This study
MuISG-15	Semi-quantitative PCR	F-5′AGCAATGGCCTGGGACCTAAA3′ R-5′AGCCGGCACACCAATCTT 3′	124	55	This study
MuIRF-7	Semi-quantitative PCR	F-5′CCCAGACTGCCTGTGTAGACG 3′ R-5′CCAGTCTCCAAACAGCACTCG 3′	71	55	This study
MuIL-4	Semi-quantitative PCR	F-5′GGTCTCAACCCCCAGCTAGT3′ R-5′GCCGATGATCTCTCTCAAGTGAT3′	102	60	This study
MuIL-6	Semi-quantitative PCR	F-5′TAGTCCTTCCTACCCCAATTTCC3′ R-5′TTGGTCCTTAGCCACTCCTTC3′	76	60	This study
MuIL-10	Semi-quantitative PCR	F-5′GCTCTTACTGACTGGCATGAG 3′ R-5′CGCAGCTCTAGGAGCATGTG 3′	105	53	This study
Influenza M-gene universal primer	Real Time quantitative PCR	F-5′ATGAGTCTTCTAACCGAGGTCGAAACG 3′ R-5′TGGACAAAGCGTCTACGCTG 3′	242	60	(Kuchipudi et al., 2012)

### Optimization of A/PR/8/1934 (H1N1) virus infection in B16F10 cells

To establish an in vitro cell culture model for testing the effect of MuIFNλ3 pre-treatment of IAV infections, we used murine B16F10 cells. In addition to expressing receptors for MuIFNλ3, B16F10 cells are also reported to be susceptible to IAV infections [25]. Prior to in vitro protection study, TCID_50_ /mL of the virus was determined for B16F10 cells. For this, the cells were grown in a 96-well plate till 80% confluency. The media was changed to fresh virus growth media consisting of incomplete DMEM media supplemented with 0.2% BSA, 1 mM MgCl_2_, 0.9 mM CaCl_2,_ and 0.5 μg/mL *N*-tosyl-l-phenylalanine chloromethyl ketone (TPCK)-trypsin (Sigma Aldrich, USA). To this, a ten-fold serially diluted A/PR/8/1934 (H1N1) virus (in PBS) was added and incubated for 1 h at 37 °C. After 1 h, the virus infection/growth media was removed, cells were washed with PBS, and fresh DMEM media was added, supplemented with 1% FBS and 0.5 μg/mL TPCK-trypsin. After 24–72 h incubation, the Reed-Muench method was employed, and the TCID_50_ /mL was calculated [10, 65].

In a parallel setup, the B16F10 cells were grown in 24-well plates till 80% confluency was reached and were subsequently infected with 0.1 MOI and 1.0 MOI of the virus as described above and incubated for the next 24 h to monitor CPEs. The percentage of cell survival against virus infection was determined by the standard MTT assay, as described in the previous section.

### Viral M-gene quantification

To check the optimal infection, influenza Type A/PR/8/1934 (H1N1) virus replication in B16F10 cells was quantified by assessing the viral M-gene transcripts using RT-qPCR. For this, the B16F10 cells were infected with 0.1 MOI and 1.0 MOI of the A/PR/8/1934 (H1N1) virus for 24 h. The TRIzol reagent was then used to extract the total RNA from the infected cells according to the manufacturer’s instructions (Invitrogen, USA). Approximately 500 ng of the total RNA was used for cDNA synthesis using the Superscript MuLV cDNA Synthesis Kit (BioBharti, India) with random hexamer primers following the manufacturer’s protocol. For M-gene quantification, 5 µL of 2 X SYBR green PCR mixture (Applied Biosystems, USA), 0.3 µL of the M-gene-specific primers, 2.4 µL nuclease-free water, and 2 µL of the 1:2 dilution of the cDNA template were mixed. The PCR conditions were set at one cycle at 50 ℃ for 2 min, 95 ℃ for 2 min, followed by 40 cycles of 95 ℃ for 15 s and 60 ℃ for 1 min. The M-gene transcript number of each group was calculated from Ct value using the standard curve plotted for the M-gene cloned into the pMD20 vector. For the standard curve, Ct values were plotted against the viral M-gene transcript number (n_molecules_) as per the formula:

$$\mathrm{n}_\mathrm{molecules}=\frac{{\mathrm{m}}_{\mathrm{template }}\times{\mathrm{ N}}_{\mathrm{A}}}{\mathrm{k }\times{\mathrm{ N}}_{\mathrm{bases}}\times{ 10}^{9}}$$

where m_template_ [ng] = quantity of the M-gene plasmid, N_bases_ [bp] = fragment length of the M-gene, k = average mass of one base (340 [Da/bp]), and N_A._ = Avogadro constant [mol^−1^] [10, 66].

### Indirect immunofluorescence assay (IFA) to detect viral nucleoprotein 

To detect viral NPs in the infected cells, IFA was carried out using the rabbit polyclonal anti-influenza NP antibody (Sino Biologicals, Japan). Briefly, B16F10 cells were grown on coverslips inside the 24-well plate and infected with the virus as described in the earlier section. Finally, the cells were washed and fixed with 4% paraformaldehyde (PFA) for 15 min at RT, then permeabilized with ice-cold acetone for 30 min. Fixed cells were incubated with a blocking solution (3% BSA in PBS-T) for 1 h at 37 ℃ and treated with the anti-NP antibody in a 1:1000 ratio. After 24 h, the cells were then incubated for 1 h at RT in the dark with FITC-labelled anti-rabbit IgG (H&L, 1:1000 dilution; Invitrogen, USA). Following washing with PBS-T and counter-staining with 4′,6-diamidino-2-phenylindole-dihydrochloride (DAPI), the cells were mounted on a glass slide using VectaShield mounting media (Vector Laboratories, USA). All images were visualized in the Leica SP8 confocal microscope using oil immersion ×63 objective (NA 1.4), and the LAS-X software was then used to acquire and process the images.

### Assessing in vitro antiviral effect of MuIFNλ3 protein secreted by r*L. lactis* cells

To determine the antiviral effect of the rMuIFNλ3, B16F10 cells (5 × 10^4^ /well in the 24-well plate) were co-cultured with r*L. lactis* in a transwell plate system, as described previously (Fig. 7). Subsequently, the cells were infected with influenza A/PR/8/1934 (H1N1) virus using 1.0 MOI of the virus by following the protocol described earlier. After 36–48 h incubation at 37 °C, the cells were processed for MTT assay, quantification of viral M-gene transcript, and IFA for detecting viral NP as per the methods described in the previous section.

Further, a viral haemagglutination (HA) assay was performed to evaluate the residual viral titer in the culture supernatant of B16F10 cells. In brief, a two-fold serial dilution of the culture supernatant (in PBS) was prepared, and 50 µL of each dilution was dispensed into a 96-well “U” bottom culture plate. After that, 50 µL of 0.5% fresh chicken erythrocyte solution (cRBC) (supplemented with 5% heat-inactivated FBS) was added to each well, and the plate was incubated for 30 min at RT. The reciprocal of the highest dilution of the sample that shows complete hemagglutination (button formation) was considered as the HA titer (HAU/50 μL) [62, 63].

### Tissue-specific immune responses in mice mucosally administered with live r*L. lactis* cells expressing MuIFNλ3

To determine the effect of mucosal administration of live r*L. lactis* cells expressing MuIFNλ3, 6–8 weeks old female BALB/c mice were divided randomly into four groups: Group-1: Control (received PBS only), Group 2: WT *L. lactis* (received live WT *L. lactis* cells), Group 3: r*L. lactis* (received live r*L. lactis* expressing MuIFNλ3) Group 4: rMuIFNλ3 (received a subcutaneous injection of IFA-emulsified rMuIFNλ3 protein purified from *E. coli*). Further details of the dose and immunization schedules are provided in Fig. 6a and Table 3.

**Table 3:** Details of different experimental groups of mice for in vivo study

Experimental group (n = 8)	Treatment details	Route of Administration
PBS	100 µL of PBS/mice	Oral + nasal (50 µL each route)
WT *L. lactis* (1 × 10^9^ CFU/mice)	Empty *L. lactis* (NZ9000) in 100 µL PBS/mice	Oral + nasal (50 µL each route)
r*L. lactis* (1 × 10^9^ CFU/mice)	Nisin-induced r*L. lactis* harbouring pSec-MuIFNλ3 plasmid in 100 µL PBS/mice	Oral + nasal (50 µL each route)
rMuIFNλ3 (1 μg/mice)	Freund’s Adjuvant (FA) emulsified purified rMuIFNλ3 protein (expressed in *E. coli*)	Subcutaneously (100 µL)

### Preparation of r*L. lactis* cells for mucosal (oro-nasal) administration

The r*L. lactis* cells were grown in M17 medium supplemented with 0.5% (w/v) glucose and 20 μg/mL chloramphenicol at 30 °C under static conditions till OD_600_ ~ 0.3. The culture was induced with 12 ng/mL nisin and grown for ~ 3 h. For the vector control, WT *L. lactis* cells were used. The nisin-induced cells were harvested by centrifugation at 2400×*g* for 5 min and washed 2–3 times with PBS. The number of cells was adjusted to 1 × 10^9^ CFU/mL in 100 μL of PBS and administered oro-nasally to the mice for three consecutive days over 3 weeks. For systemic administration, 1 μg of IFA-emulsified rMuIFNλ3 protein was administered subcutaneously on indicated time points, followed by secondary administration on days 14 and 21 with 1 µg of IFA-emulsified rMuIFNλ3 protein.

### Assessing the in vivo viability of r*L. lactis*

To check the in vivo viability of the modified vector harboring recombinant plasmid, fresh fecal samples were collected from each study group and processed as per the established protocols with some modifications [67]. In brief, homogenized fecal pellets were plated onto an M17 agar plate supplemented with 0.5% glucose and with or without 20 µg/mL chloramphenicol and incubated at 30 °C in static conditions. Colonies that appeared in the next 24 h were randomly selected and subjected to colony PCR using MuIFNλ3-specific primers set (Table 2).

### Transcriptional analysis of antiviral genes in lung and intestinal tissue

For transcriptional analysis, on day 7 post-last treatment, mice from the different treatment groups were euthanized by CO_2_ inhalation, and approximately 0.10 gm of the lung and intestine tissues were aseptically collected. For RNA extraction, the tissues were thoroughly washed with 1X PBS, minced, and the total RNA was extracted using the TRIzol method, per the manufacturer’s instructions (Invitrogen, USA). For each sample, 1000 ng of total RNA in a final volume of 20 μL reaction mixture was transcribed to cDNA using the Superscript MuLV cDNA Synthesis Kit (BioBharati, India). The transcriptional analysis was performed by semi-quantitative PCR for MX-1, ISG-1, IRF-7, IL-4, IL-6, and IL-10 genes, and MuGAPDH was taken as an endogenous control.

### Histopathological assessment of lung and intestinal tissue of experimental mice

To see the effect of MuIFNλ3 on lung or intestinal tissue, on day 28, approximately 0.10 gm of the tissue was collected from the different treatment groups and subjected to histopathological analysis. Briefly, the tissue was sliced to a thickness of ~ 0.5 cm and fixed in a 10% formalin solution. Then, the sections were washed under running water and dehydrated using an ascending acetone gradient (70%, 90%, and 100%). The dehydrated tissues were cleaned and methodologically impregnated with melted paraffin at 62 ℃. The paraffin blocks were further sectioned and proceeded for H & Estaining [23].

### Statistical analysis

The experimental data of the gene expression study was analyzed for any significant difference by calculating the band intensity for each gene using ImageLab software (Version 3.0.1). The GraphPad Prism software (Version 8) was used to plot the graphs and analyze the experimental data using the non-parametric Mann–Whitney U test. A *p*-value less than 0.05 (*p* < 0.05) was considered statistically significant.

### Supplementary Information

**Additional file 1: Table S1.** Summary of transcriptional profiles of target genes (in vitro and in vivo studies).** Figure S1.** Determination of CC_50_ of rMuIFNλ3 protein in murine J774A.1 cells. **Figure S2.** Quantification of sMuIFNλ3 in the culture supernatant of *rL. lactis* by ELISA. **Figure S3.** Semi-quantitative RT-PCR of the target genes in response to MuIFNλ3 pre-treatment. **Figure S4.** Retrieval of r*L. lactis* from the experimental mice. **Figure S5.** Histopathological and transcriptional analysis of the small intestinal tissues.

## Data Availability

All data generated or analyzed during this study are included in this published article [and its supplementary files].
