# Supplementary material for: The immune-adjunctive potential of recombinant LAB vector expressing murine IFNλ3 (MuIFNλ3) against Type A Influenza Virus (IAV) infection
Source: Gut Pathog. 2023 Oct 30;15:53. doi: 10.1186/s13099-023-00578-5 (PMC10617148; doi:10.1186/s13099-023-00578-5)
Supplement: Supplementary file 1 — Additional file 1: Table S1. Summary of transcriptional profiles of target genes (in vitro and in vivo studies). Figure S1. Determination of CC50 of rMuIFNλ3 protein in murine J774A.1 cells. Figure S2. Quantification of sMuIFNλ3 in the culture supernatant of rL. lactis by ELISA. Figure S3. Semi-quantitative RT-PCR of the target genes in response to MuIFNλ3 pre-treatment. Figure S4. Retrieval of rL. lactis from the experimental mice. Figure S5. Histopathological and transcriptional analysis of the small intestinal tissues. [file 13099_2023_578_MOESM1_ESM.docx]

The immune-adjunctive potential of recombinant LAB vector expressing murine IFNλ3 (MuIFNλ3) against Type A Influenza Virus (IAV) infection

Sandeep Yadav^a#^, Aparna Varma ^a#^, Aparna Odayil Muralidharan ^a#^, Sucharita Bhowmick^a^, Samiran Mondal^b^ and Amirul Islam Mallick ^a*^

^a^Department of Biological Sciences, Indian Institute of Science Education and Research Kolkata, Mohanpur, Nadia, 741246, West Bengal, India.

^b^Department of Veterinary Pathology, West Bengal University of Animal and Fishery Sciences,

Kolkata, 700037, West Bengal, India

^#^ Authors contributed equally

***Corresponding Author**

Dr. Amirul Islam Mallick, Associate Professor, Department of Biological Sciences, Indian Institute of Science Education and Research Kolkata, Mohanpur, Nadia, West Bengal, 741246, India. Ph. 91-33-61360022-Ext 1221, E-mail: [amallick@iiserkol.ac.in](mailto:amallick@iiserkol.ac.in)

**Table S1.** **Summary of transcriptional profiles of target genes (*in vitro* and *in vivo* studies)**

| Gene | Function | *In vitro* transcriptional profile (fold changes)  (B16F10 cells) | *In vivo* transcriptional profile  (fold changes) | |
| --- | --- | --- | --- | --- |
|  |  |  | **Lungs** | **Intestine** |
| **MX-1** | GTPase | **Upregulated**  (1.20)  (*p*<0.0286) | **Upregulated**  (1.12)  (*p*<0.0057) | **Unchanged**  (1.04)  (*p*>0.3143) |
| **ISG-15** | Interferon Stimulatory Gene | **Upregulated**  (1.28)  (*p*<0.0306) | **Upregulated**  (1.14)  (*p*<0.0002) | **Unchanged**  (1.05)  (*p*>0.9999) |
| **IRF-7** | Interferon Regulatory Factor | **Upregulated**  (1.20)  (*p*<0.0286) | **Upregulated**  (1.15)  (*p*<0.0002) | **Unchanged**  (1.01)  (*p*>0.3143) |
| **IL-6** | Pro-inflammatory cytokine | **Upregulated**  (1.11)  (*p*<0.0023) | **Upregulated**  (1.10)  (*p*<0.0243) | **Unchanged**  (1.01)  (*p*>0.9999) |
| **IL-10** | Anti-inflammatory cytokine | **Upregulated**  (1.12)  (*p*<0.1321) | **Upregulated**  (1.16)  (*p*<0.0057) | **Unchanged**  (1.12)  (*p*>0.3143) |
| **IL-4** | Anti-inflammatory cytokine | **Upregulated**  (1.15)  (*p*<0.0731) | **Unchanged**  (0.96)  (*p*<0.4124) | **Unchanged**  (1.01)  (*p*<0.3143 |

**Figure S1: Determination of CC_50_ of rMuIFNλ3 protein in murine J774A.1 cells**

**Fig. S1.** The CC_50_ value of the rMuIFNλ3 protein was calculated in murine J774A.1 macrophage cells and was estimated to be >100 µg/mL. Each point represents the mean of two independent data sets ± the standard deviation (SD) of the means.

**Figure S2. Quantification of secretory MuIFNλ3 (sMuIFNλ3) protein in the culture supernatant of r*L. lactis* by indirect ELISA**

**b**

**a**

**Fig. S2:** Standard curve generated by indirect ELISA using a known concentration of rMuIFNλ3 protein purified from r*E. coli* (Log_10_ ng/mL) **(a)**. To quantify the rMuIFNλ3 protein secreted by r*L. lactis,*  TCA precipitated protein fraction from the culture supernatant of nisin-induced r*L.lactis* cells grown at a different density were subjected to indirect ELISA. The mean absorbance (A_450_) for cell density was plotted against the protein standard concentration, suggesting approximately ~2.5 ng/mL of protein produced by 1 × 10^9^ r*L. lactis* cells at 3 h post-induction **(b)**.


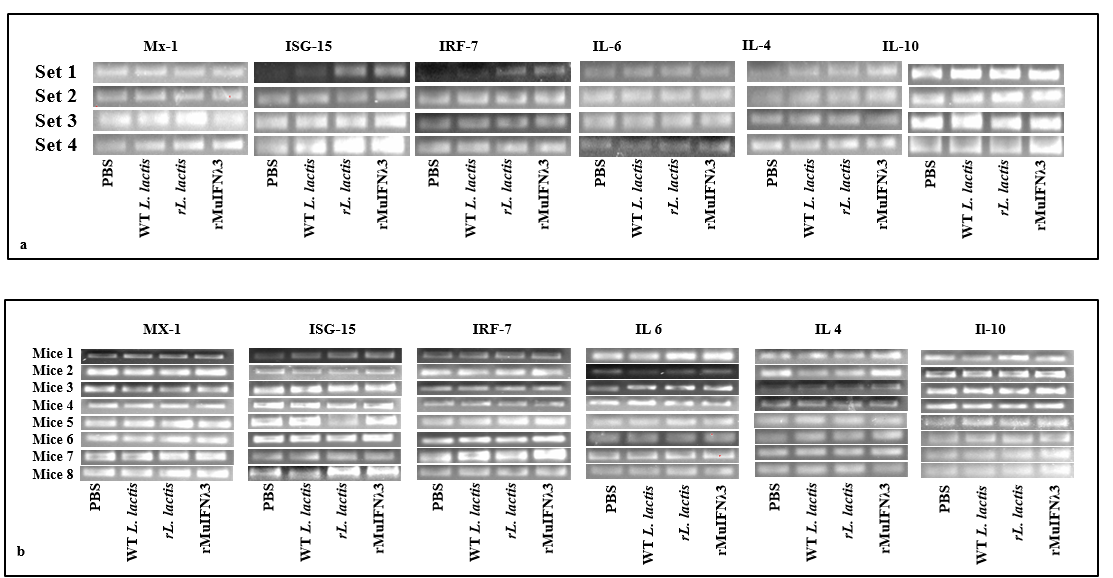


**a**

**b**

**IL-4**

**IL-10**

**IL-6**

**b**

**a**

**Figure S3. Semi-quantitative PCR of the target genes in response to MuIFNλ3 pre-treatment**

**Fig. S3:** Gel images of target gene expressed in B16F10 cells pre-treated with different forms of MuIFNλ3 protein **(a)**. Gel images of target genes expressed in lung tissue of mice administered with different forms of MuIFNλ3 protein **(b)**. After normalization of each gene by densitometry using mice GAPDH gene as an internal control, semi-quantitative RT-PCR was performed for each target gene.

**Figure S4: Retrieval of r*L. lactis* from the experimental mice**

**Fig. S3.** Agarose gel images of target gene expressed in B6F10 cells pre-treated with different forms of MuIFNλ3 protein **(a)**. Agarose gel images of target genes expressed in lung tissue of mice administered with different forms and routes of MuIFNλ3 protein **(b)**. After quantitative normalization for each gene by densitometry using murine GAPDH gene expression, semi-quantitative PCR was performed for the target genes.

**Figure S4. Retrieval of r*L. lactis* from the experimental mice**


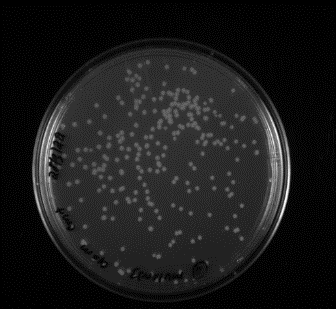

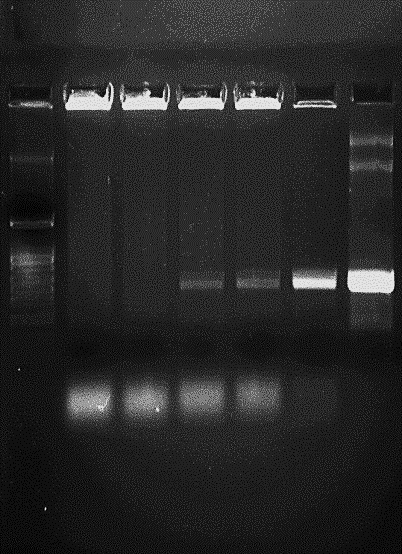


**M 1 2 3 4 5 6**


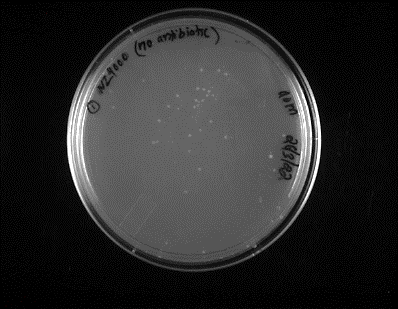


**WT *L. lactis***

(**M17 agar plate)**


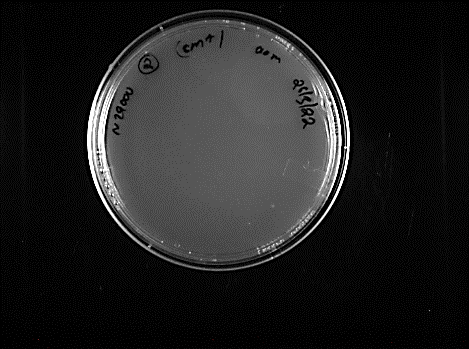


**WT *L. lactis***

(**M17 agar plate**

**with chloramphenicol)**

**r*L. lactis***

(**M17 agar plate**

**with chloramphenicol)**

**a**

**b**

**c**

**d**

**Fig. S4.** *In vivo* survivability of *L. lactis* in experimental mice was checked by its retrieval from fresh fecal samples collected from the experimental animal. In the case of WT *L. lactis* administered mice, colonies appeared when grown in the absence of chloramphenicol **(a)**; however, no colonies could be seen in the plate containing chloramphenicol (a selective antibiotic marker for the plasmid) **(b).** Fecal samples from the mice were administered with r*L. lactis,* when cultured in the plates containing chloramphenicol, show characteristic milky white colonies suggesting bacterial survival during *in vivo* gut transit **(c).** Colony PCR of randomly picked colonies from the plate **‘c’ (**r*L. lactis* administered mice) further confirmed the presence of intact plasmid encoding the target gene corresponding to the size of MuIFNλ3 (lane 3-5), while no amplification was seen when colonies picked from the plate **‘a’** (WT *L. lactis*) (lane 1,2). (M: Marker, lane 6: Positive control-pSec-MuIFNλ3 plasmid as DNA template) **(d).**


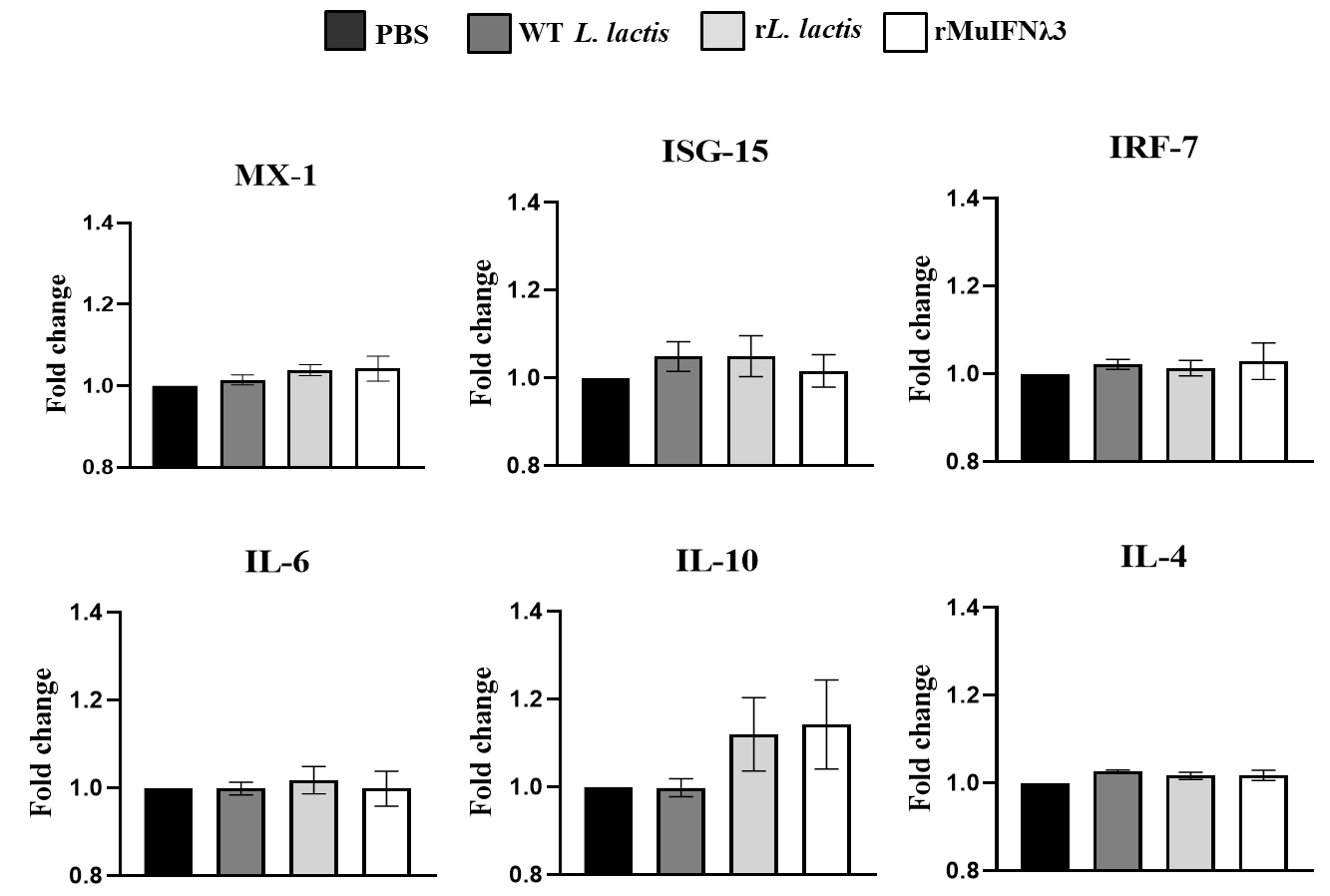
**F****igure S5: Histopathological and transcriptional analysis of the small intestinal tissues**

**b**

**a**


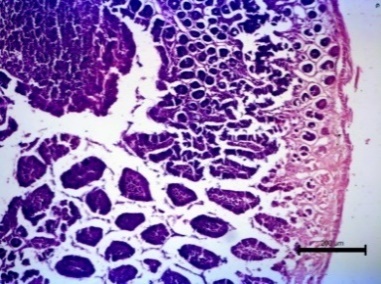

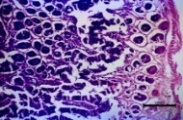

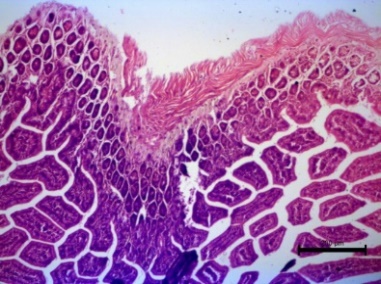

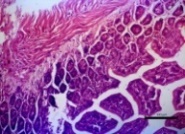

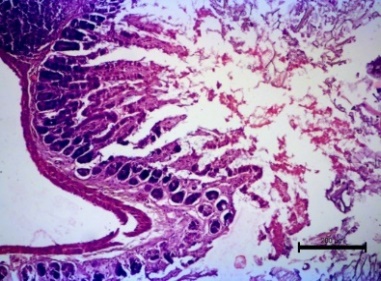

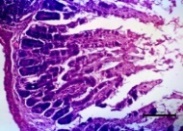

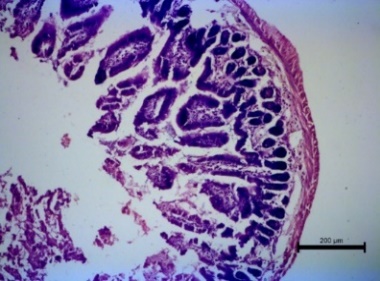

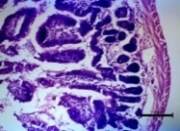


**PBS**

**WT *L. lactis***

**r*L. lactis***

**rMuIFNλ3**

**Fig. S5.** Representative images of intestinal tissue (duodenum) section stained with H&E. Histo-morphological analysis of tissue sections collected from PBS-administered mice show typical tissue architecture characterized by the intact outer longitudinal muscular layer and inner circular muscle over a thin serosal layer. In WT *L. lactis* administered (oro-nasally) mice, the mucosal layer shows mild degenerative changes in the columnar epithelium with minor congestion of the blood vessels in the sub-mucosal layer and infiltration of mononuclear cells. Mice that received r*L. lactis* showed mild hyperplasia of the outer longitudinal and inner circular muscle layer. Mild congestion of the blood vessels is also noticeable in the lamina propria. In the group administered with rMuIFNλ3, the disruption in the outer muscular layers with multi-focal mononuclear infiltration in the submucosal layer could be seen. The mucosal layer revealed some disruption of the epithelial layer of the villi with mild loss of architectural details of the epithelium **(a)**. *In vivo* gene expression of immune-regulatory and antiviral genes in response to MuIFNλ3 pre-treatment in the intestinal tissue of mice **(b).**
